# Long multiply marked DNA repair template reveals lengths and fidelity of genome editing tracts in *Schizosaccharomyces pombe*

**DOI:** 10.17912/micropub.biology.001917

**Published:** 2025-11-13

**Authors:** Reine U Protacio, Nyera A Ali, Akshara Chevireddy, Wayne P Wahls

**Affiliations:** 1 Biochemistry and Molecular Biology, University of Arkansas for Medical Sciences, Little Rock, Arkansas, United States; 2 Pulaski Academy High School, Little Rock, Arkansas, United States

## Abstract

To test the ability of a fission yeast CRISPR-Cas9 system ( *SpEDIT* ) to carry out genome editing over distance, we constructed a 1,935 bp-long, dsDNA repair template that contained 45 base pair substitutions (SNPs), relative to the wild-type target locus *ade6* . Template-directed repair was efficient in the vicinity of the recombination-initiating dsDNA break, but the efficiency fell rapidly with distance (median editing tract length of 163 bp). The regularly distributed markers also revealed evidence for heteroduplex DNA at the ends of repair tracks and, unexpectedly, that DNA ends of the repair template participate in many (~18%) of the genome editing events.

**Figure 1. Lengths and fidelity of genome editing tracts:**
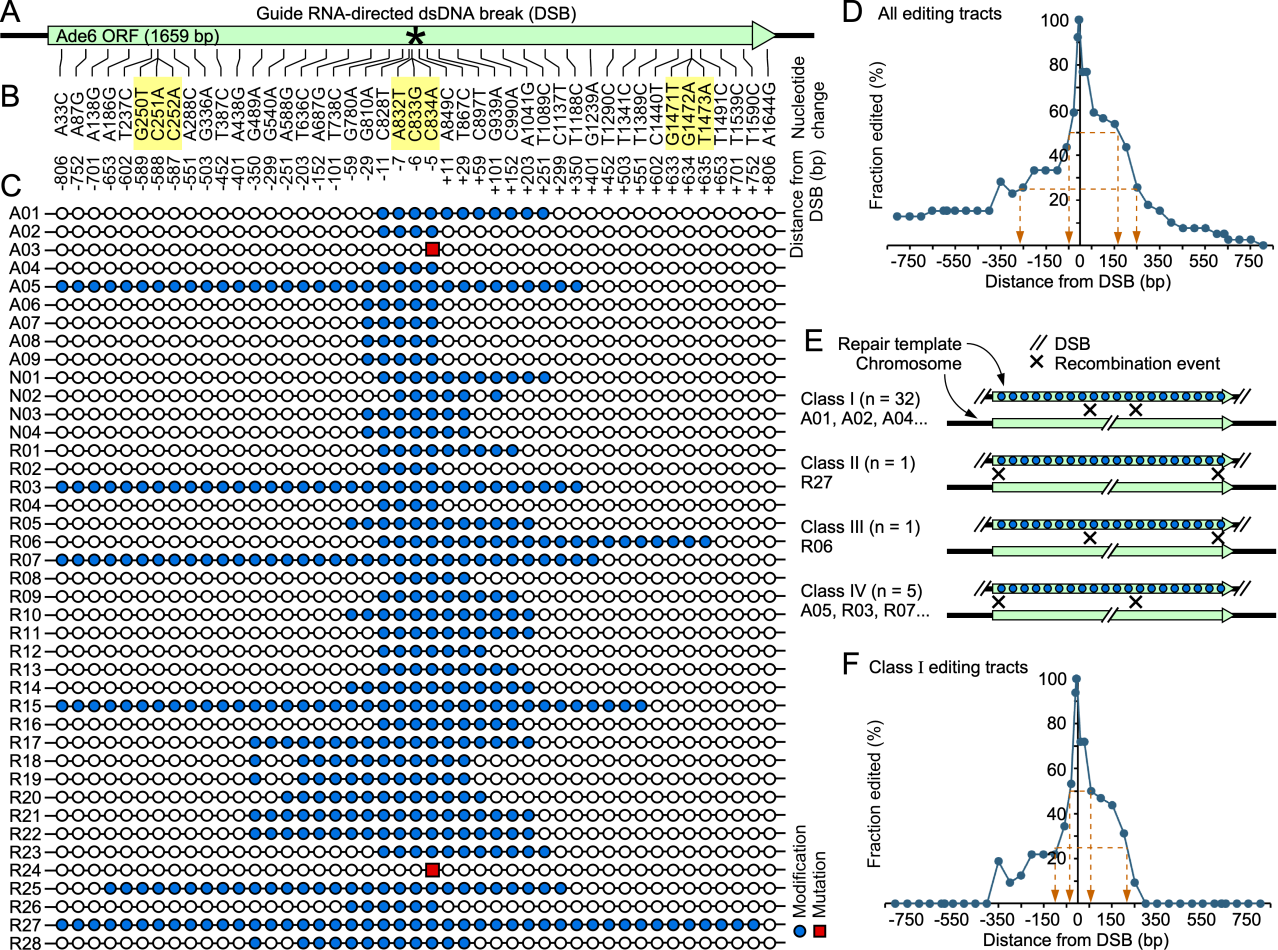
**A.** A recombination-initiating DSB was induced at the center (*) of the *ade6* ORF in the chromosome. **B.** A 1,935 bp-long, linear, homologous, dsDNA repair template contained 45 base pair substitutions (nucleotide positions are numbered relative to +1 of the ATG); highlighted changes ( *yellow* ) create stop codons. Distances from position of the DSB are also indicated. **C.** Each line depicts DNA sequence results for an individual clone. Circles represent (and are aligned with) substitutions listed in panel B; filled circles ( *blue* ) indicate successful template-directed modifications; squares ( *red* ) denote mutations from NHEJ. **D.** Plot shows distance-dependent decay in frequency of editing; distances at which editing drops below 50% and 25% are highlighted ( *orange arrowheads* ). **E.** Recombination-initiating DNA ends can be provided by the DSB in the chromosome or by the termini of the repair template, supporting four classes of recombination-dependent editing events. **F.** Distance-dependent decay in frequency of editing for the predominant Class I events.

## Description

Precise genome editing in the fission yeast *S. pombe* has historically been achieved using two steps of gene targeting (Grimm *et al.* 1988) or a highly efficient pop-in, pop-out strategy (Gao *et al.* 2014). Subsequently developed, CRISPR-Cas9-based editing systems, such as *SpEDIT* (Torres-Garcia *et al.* 2020), use a synthetic guide RNA (sgRNA) and Cas9 endonuclease to direct a recombination-initiating dsDNA break (DSB) to a specific site in the genome; then, the DSB is repaired by recombination with a linear homologous recombination (HR) template DNA molecule (HR template). *SpEDIT* using HR templates 180 bp in length reportedly yielded a 100% efficiency for introducing bp substitutions into *ade6* and *ura4* (Torres-Garcia *et al.* 2020). However, subsequent studies with similarly sized templates revealed that this high efficiency was restricted to bp changes close to the DSB; the editing tracts within *ade6* and *ura4* frequently did not extend to bp substitutions placed about 50 bp away (Protacio *et al.* 2024a; Protacio *et al.* 2024b). This raised questions about the utility of the approach for larger tracts of editing. We therefore developed a way to measure with precision the lengths and fidelity of genome editing tracts over much longer distances.

We used *SpEDIT* to introduce a DSB at the center of the *ade6* ORF ( **Figure 1A** ) and we provided an HR template molecule 1,935 bp in length ( **Figure 1B** ). The HR template contained 45 single bp substitutions ( *aka* SNPs) distributed over 1,612 bp of the *ade6* ORF. Three substitutions adjacent to the position of the DSB (A832T, C833G, C834A) were designed to simultaneously create a stop codon (for diagnostic screening) and to inactivate the PAM motif (to prevent multiple cycles of endonucleolytic cleavage after the first round of editing). The rationale, based on previous findings (Protacio *et al.* 2024a; Protacio *et al.* 2024b), is that recombination-based editing events should initiate at the DNA ends created by the DSB and, therefore, the highest frequency of successful editing should occur in that vicinity. Nevertheless, we also positioned two additional stop codons about 600 bp 5' and 3' of the DSB, respectively, which allowed us to score for editing events that might initiate distal to the DSB without involving any DSB-proximal editing. All other SNPs were translationally silent. Cells that were wild-type for *ade6* were cotransformed with plasmid expressing the *ade6* -targeting sgRNA-Cas9 endonuclease and the HR template, were plated on selective media with limiting adenine, and colonies were screened for the red colony phenotype that is diagnostic for mutations within *ade6* . We sequenced the *ade6* locus from 41 candidates that passed this screen, which revealed the following.

Two of the 41 clones had no template-directed bp substitutions ( **Figure 1C** ). Clone A03 had a deletion of two adjacent base pairs (A840del, G841del) and clone R24 had a single bp deletion (G841del). These small indels, adjacent to the position of the DSB, are characteristic of nonhomologous end joining (NHEJ). They indicate that the gRNA-directed DSB formed properly but the broken chromosome failed to engage productively with an HR repair template, leading to mutagenic NHEJ.

In contrast, 39 of the clones stemmed unambiguously from template-directed editing at four or more SNPs ( **Figure 1C** ). The tracts of editing ranged from as short as 7 bp (e.g., as in Clone A02) to as long as 1,558 bp (e.g., clone R27). With few exceptions (see below), the tracts were continuous but incomplete: none of the events copied all 45 SNPs from the HR template. Moreover, the mean (341 bp) and median (163 bp) extents of editing were far less than the length of the HR template (1,935 bp). Indeed, the frequency of editing a given position along the chromosome decayed rapidly as a function of distance from the recombination-initiating DSB ( **Figure 1D** ). For example, half of the repair tracts failed to extend more than ~50 bp to left of the DSB, and 75% of tracts ended within ~250 bp. This rapid decay is consistent with findings reported for the same *SpEDIT* approach using shorter (196 bp and 200 bp) HR templates (Protacio *et al.* 2024a; Protacio *et al.* 2024b).

The results also revealed, unexpectedly, two different types of polarity ( **Figure 1C** and **1D** ). First, distance-dependent decay was stronger immediately to the left of the DSB than immediately to its right. Second, there was asymmetry in the longer repair tracts: six of them had left ends that were relatively close to the left end of the HR template, whereas only two had right ends towards the right end of the HR template. The fact that DNA ends are recombinogenic (Cejka and Symington 2021) provides insight: DNA ends located on the linear HR template molecule, as well as the DNA ends flanking the DSB, should be able to initiate recombination. This principle, and our data, indicate that there are four distinct classes of editing tracts, the majority of which (Class I) are initiated by and centered around the sgRNA-positioned DSB ( **Figure 1E** ). Focusing on the Class I editing tracts demonstrated that the strong polarity of distance-dependent decay immediately adjacent to the DSB is intrinsic to this class ( **Figure 1F** ), unrelated to the HR template-end-use polarity.

The mechanisms for polarity are unknown.&nbsp; Hypothetically, polarity of editing on each side of the DSB ( **Figure 1F** ) might be imparted by the polarity of transcription:&nbsp; RNA polymerase transcribing the *ade6* gene could disrupt recombination intermediates ( *e.g.* , end invasions) just to the left of the DSB, but not translocate through the DSB to disrupt intermediates to the right.&nbsp; Hypothetically, polarity associated with the ends of the HR template DNA ( **Figure 1C** and **1D** ) might be imparted by the chromatin structure of the chromosomal *ade6* locus: The nucleosome-free region in the *ade6* promoter (Mukiza *et al.* 2019) could facilitate greater end invasion by the left end of the HR template, relative to end invasion by the right end, which would be invading more-organized chromatin.&nbsp; These hypotheses await further testing ( *e.g.* , at multiple loci that do or do not have transcription or at which transcription rates could be modulated).&nbsp; Meanwhile, the current data provide insight into other mechanisms.

Class I-type ( *i.e.,* DSB / gap repair-type) editing events involve DNA end resection flanking the chromosomal DSB, followed by template-directed refilling of the gap [see review by (Cejka and Symington 2021)]. Thus, the lengths of editing tracts provide a minimum value for the amount of resection (resection could have extend a bit farther than the location of the last informative SNPs in each edited tract). Collectively, our data for the 32 Class I events provide robust lower-bound estimates for the mean (165 bp), median (163 bp) and maximum (553 bp) amounts of dsDNA end resection—for a chromosomal DSB in mitotic fission yeast cells in the presence of a homologous DNA molecule from which to affect repair. This resection length is similar to that inferred by other means (Gao *et al.* 2014).

Processing of DSBs / gaps by 5' to 3' ssDNA end resection produces 3' ssDNA regions flanking each DSB gap (Cejka and Symington 2021). When these 3' ssDNA tails invade and base pair with a homologous DNA repair template, they form heteroduplex DNA (hDNA). Then, template-dependent DNA polymerase activity fills in the gap with homoduplex DNA whose sequence is identical to that of the template. Mismatched base pairs in the flanking hDNA can be repaired in one of two directions: they can get *restored* to match the chromosomal sequence (leaving no trace of their existence) or *converted* to that of the template sequence. Remarkably, our use of multiple, regularly spaced SNPs in the repair template allowed us to detect signatures of hDNA in about 12% of the Class I clones (N02, R18, R19, R28 in **Figure 1C** ). As predicted, the hDNA occurred exclusively towards the ends of the repair tracts. For example, in clone R28 sequential SNPs at the left side of the editing tract alternated between those of the chromosome (first letter highlighted) or template (second letter highlighted): **A** 438G, G489 **A** , **G** 540A, **A** 588G, T636 **C** . This pattern indicates that hDNA spanned this region and that there were at least two patches of MMR operating on opposite DNA strands, with maximum possible lengths of 103 bp and 148 bp, respectively. Overall, based on eight informative patches of MMR, our data revealed upper limits for the range (73 bp to 148 bp) and mean (115 bp) lengths of MMR.

In conclusion, the long, multiply marked DNA repair template revealed at high resolution the lengths and fidelity of CRISPR-Cas9-mediated genome editing in fission yeast. Although the editing tracts are largely continuous, their short lengths (163 bp median) limit the utility of this approach for many applications. The unexpected involvement of HR template ends in a subset of events can contribute to longer editing tracts, although those are mainly asymmetric. We posit that the use of two sgRNA-directed DSBs in the genome, positioned towards ends a long HR template molecule, might improve efficiency. Alternatively, one could choose to use pop-in, pop-out allele replacement, which can rapidly, efficiently and precisely edit thousands of base pairs in the fission yeast genome (Gao *et al.* 2014; Storey *et al.* 2019).

## Methods

We used published protocols for the fission yeast-optimized, genome editing system known as *SpEDIT* (Torres-Garcia *et al.* 2020; Protacio *et al.* 2024a). The CRISPR4P program (Rodriguez-Lopez *et al.* 2016) was used to design the optimal sgRNA for our *ade6* target. Oligonucleotides ade6 sgRNA-F and ade6 sgRNA-R (see **Reagents** / Oligonucleotides) were annealed and cloned into plasmid pLSB- *kanMX6* to encode a synthetic guide RNA (sgRNA116) targeted to the center of the *ade6* ORF. To create the HR repair template, an *ade6* gene that contained 45 different bp substitutions was synthesized as a GBlock (Integrated DNA Technologies) and was cloned via *Sa* cI and *Ps* tI linkers into pUC19 to generate pUC19- *ade6 smts* (see **Reagents** / Plasmids). This clone bears 1,935 bp of homology to the *ade6* locus (143 bp of 5' sequence; 1,659 bp of ORF; 133 bp of 3' sequence). After plasmid amplification, the *Sa* cI- *Ps* tI fragment bearing the *ade6-smts* gene was excised, gel purified, and used as the HR template. The procedures for genome editing were as described (Protacio *et al.* 2024a). In brief, fission yeast strain WSP 3776 (see **Reagents** / Fission yeast strains) was cotransformed with pLSB- *kanMX6* -sgRNA116 and the repair template *ade6-smts* , cells were plated on YEA media that contained G418 and were incubated at 32 °C for 4 days to select for transformants, and red colonies (a phenotype generated by mutations in *ade6* ) were studied further. These clones were streaked on nonselective media to permit loss of the CRISPR-Cas9-encoding plasmid. Individual clones were then each cultured overnight in 5 ml of YEL (liquid) culture media, a smash and grab method was used to prepare genomic DNA (Hoffman 2001), and the genomic DNAs were subject to PCR amplification using primers Gblock-F and Gblock-R (see **Reagents** / Oligonucleotides). Oligonucleotides Gblock-F, ade6-15F, ade6-19L, and Gblock-R were used to sequence the *ade6* locus of each clone; then, those sequences were compared to that of wild-type *ade6* to determine which of the 45 different SNPs in the HR repair template had been successfully edited into the genome of each clone (depicted in **Figure 1C** ).

## Reagents

**Table:** 

Oligonucleotides:	
**Name**	**Sequence**
ade6 sgRNA-F	5'-CTAGAGGTCTCGGACTAGAAGTTGGGCAAGCTTC AAGTTTCGAGACCCTTCC-3'
ade6 sgRNA-R	5'-GGAAGGGTCTCGAAACTTGAAGCTTGCCCAACTTCT AGTCCGAGACCTCTAG-3'
Gblock-F	5'-CGATGCAAAACTCAAATAATAAACTGCG-3'
Gblock-R	5'-CACTTTTTAGAATACATTTTACAATCTAGAATTTC-3'
ade6-15F	5'-ATGCTTATCCTACAACTGAGACC-3'
ade6-19R	5'-GCAGCATCTTTCATCTTGCTT-3'

**Table:** 

Fission yeast strains:		
**Name**	**Genotype**	**Source**
WSP 3776	*h- wildtype*	Wahls Lab

**Table:** 

Plasmids:		
**Name**	**Genotype**	**Notes**
pUC19- ade6 smts	*ade6-A33C, A87G, A138G, A186G, T237C, G250T, C251A, C252A, A288C, G336A, T387C, A438G, G489A, G540A, A588G, T636C, A687G, T738C, G780A, G810A, C828T, A832T, C833G, C834A, A849C, T867C, C897T, G939A, C990A, A1041G, T1089C, C1137T, T1188C, G1239A, T1290C, T1341C, T1389C, C1440T, G1471T, G1472A, T1473A, T1491C, T1539C, T1590C, A1644G*	The *ade6* gene was cloned between *Sa* cI and *Ps* t1 restriction sites of pUC19; positions of the engineered SNPs are numbered relative to +1 of the *ade6* ORF (e.g., A33C is an A to C substitution at nucleotide position 33 of the ORF).

**Table:** 
